# Human Bartonellosis: An Underappreciated Public Health Problem?

**DOI:** 10.3390/tropicalmed4020069

**Published:** 2019-04-19

**Authors:** Mercedes A. Cheslock, Monica E. Embers

**Affiliations:** Division of Immunology, Tulane National Primate Research Center, Tulane University Health Sciences, Covington, LA 70433, USA; mchesloc@tulane.edu

**Keywords:** *Bartonella*, vector, bartonellosis, ticks, fleas, domestic animals, human

## Abstract

*Bartonella* spp. bacteria can be found around the globe and are the causative agents of multiple human diseases. The most well-known infection is called cat-scratch disease, which causes mild lymphadenopathy and fever. As our knowledge of these bacteria grows, new presentations of the disease have been recognized, with serious manifestations. Not only has more severe disease been associated with these bacteria but also *Bartonella* species have been discovered in a wide range of mammals, and the pathogens’ DNA can be found in multiple vectors. This review will focus on some common mammalian reservoirs as well as the suspected vectors in relation to the disease transmission and prevalence. Understanding the complex interactions between these bacteria, their vectors, and their reservoirs, as well as the breadth of infection by *Bartonella* around the world will help to assess the impact of Bartonellosis on public health.

## 1. Introduction

Several *Bartonella* spp. have been linked to emerging and reemerging human diseases (Table 1) [1,2,3,4,5]. These fastidious, gram-negative bacteria cause the clinically complex disease known as Bartonellosis. Historically, the most common causative agents for human disease have been *Bartonella bacilliformis, Bartonella quintana,* and *Bartonella henselae*. These infections cause a variety of manifestations from mild symptoms such as fever, headache, and malaise to more severe symptoms such as hallucinations [3,5,6,7,8].

**Figure 1:**
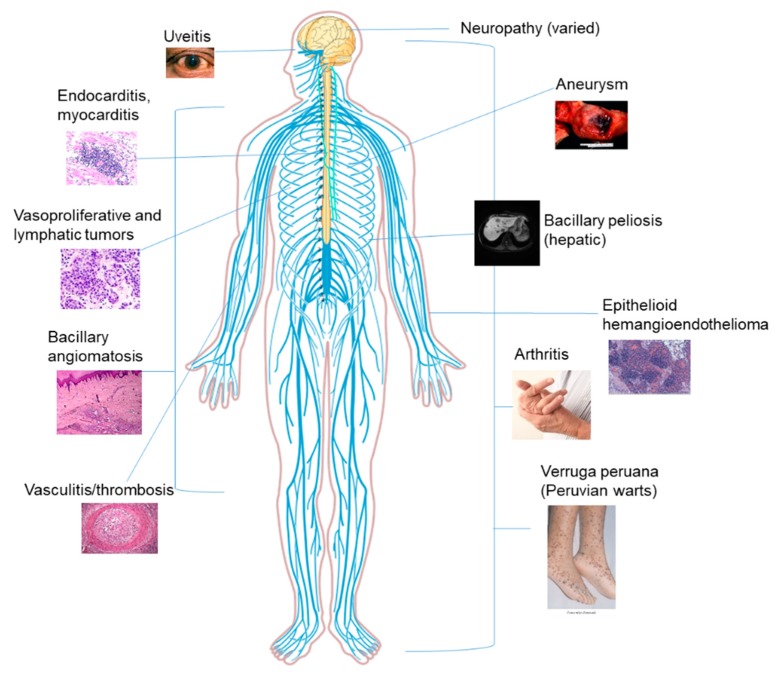
The clinical manifestations of human Bartonellosis.

Bartonellosis is characterized by a prolonged intraerythrocytic bacteremia within a diverse array of reservoirs hosts [1]. *Bartonella* spp. have been isolated from numerous hosts including humans, cats, dogs, rabbits, rodents, horses, cattle, and other wild animals [3,4,5]. The severity of the clinical manifestations is often correlated with the immune status of the patient, although other factors such as the species infecting the host, virulence factors, and bacterial load should be considered as components in disease severity. These features allow *Bartonella* spp. to persist in the blood of hosts as a chronic infection and to account for the range of clinical manifestations (Figure 1) [1]. Known diseases caused by *Bartonella* infections include Carrion’s disease, cat-scratch disease, chronic lymphadenopathy, trench fever, chronic bacteraemia, culture-negative endocarditis, bacilliary angiomatosis, bacilliary peliosis, vasculitis, and uveitis [1,2,4,6,7,9,10,11]. Recently, Bartonella infections have been linked to more diverse manifestations such as hallucinations, weight loss, muscle fatigue, partial paralysis, pediatric acute-onset neuropsychiatric syndrome (PANS), and other neurological manifestations [6,8,10]. A few case studies have also documented *Bartonella* in tumors, particularly vasoproliferative and those of mammary tissue [12,13,14]. The potential involvement of this pathogen in breast tumorigenesis is both disconcerting and warrants significantly more research. *Bartonella* spp. are zoonotic pathogens transmitted from mammals to humans through a variety of insect vectors including the sand fly, cat fleas, and human body louse [4,5]. New evidence suggests that ticks, red ants, and spiders can also transmit *Bartonella* [15,16,17,18]. Bed bugs have been implicated in the transmission cycle of *B. quintana* and have been artificially infected [19]. *B. quintana* was found in bed bug feces for up to 18 days postinfection [19]. The diversity of newly discovered *Bartonella* species, the large number and ecologically diverse animal reservoir hosts, and the large spectrum of arthropod vectors that can transmit these bacteria among animals and humans are major causes for public health concern. 

## 2. Bartonella in Domestic Animals and the Potential for Transmission

### 2.1. Cats

Cats become infected with several species of *Bartonella*, yet the symptoms of these infections widely vary. In 1995, evidence established that cats were the main reservoir for *Bartonella henselae*, the causative agent of cat-scratch disease [20]. Cat-scratch disease is typically a self-limiting but long-lasting swelling of the draining lymph nodes from the site of infection. Patients present with regional lymphadenitis about 3–10 days after the cat scratch, and the papule will last anywhere from a few days to 2–3 weeks [1]. 

Cats have also been discovered as reservoirs for other *Bartonella* species such as *B. clarridgeiae* and *B. koehlerae* [9,21,22]. *B. clarridgeiae* and *B. koehlerae* are both causative agents of the cat-scratch disease-like illness. Of significance, *B. koehlerae* has been associated with serious disease [1,22] including endocarditis and neuropathy [1,18,23,24]. *Bartonella* endocarditis classically presents in patients with preexisting heart valve conditions and results in blood-culture-negative endocarditis [23]. A neurological disease has been reported in multiple case studies [8,18,24]. In one case study, a woman experiencing depression, anxiety, mood swings, severe headaches, and hallucinations was diagnosed with Bartonellosis through an immunofluorescent antibody detection assay. Once the patient was treated with antibiotics, her neurological symptoms ceased [8]. To determine cats’ capacity as a reservoir for multiple *Bartonella* species, one study was conducted whereby specific pathogen-free cats were experimentally infected with *B. vinsonii* subsp. *berkhoffii*, *B. quintana*, *B. bovis*, *B. weissii* (feline isolate of *B. bovis*), or *B. rochalimae* [25]. The study determined that cats may be a reservoir of *B. rochalimae* but did not appear to be reservoirs of the remaining *Bartonella* species [25]. The authors did note that the cats were not infected through a vector, and other factors could contribute to bacteremia in the wild, making wild cats more susceptible to infections by *Bartonella* species [25].

Cats infected with *Bartonella* usually have asymptomatic bacteremia that can last for many months. A transmission to humans occurs directly from a cat scratch and may occur through the cat flea *Ctenocephalides felis* [3,9,26,27]. There has been some debate on whether the bacteria can be transmitted from a cat’s bite. *B. henselae* DNA has been isolated from cat saliva, although there has been no evidence that a cat bite has led to cat-scratch disease directly [28]. Also, in a case report from 2006, a woman was bitten by a cat and identical *Bartonella quintana* DNA was detected from both the woman and the cat that bit her; however, the woman admitted to daily exposure to biting flies and mosquitoes, with an occasional exposure to ticks and fleas, as well as having been bitten by a dog earlier that day, so the source of the bacteria could not be determined [29]. A transmission between cats through *C. felis* has been shown experimentally [26]. Some new evidence also indicates that ticks can transmit *B. henselae.* In this case, *Ixodes ricinus* ticks removed from a cat that had anti-*B. henselae* IgG antibodies also tested positive by PCR for *B. henselae* DNA [30]. Although there is supportive evidence, a competence study using ticks and *B. henselae* must be performed to definitively claim that ticks are a vector in transmitting this pathogen.

### 2.2. Dogs

Dogs have been infected with *B. henselae*, B. vinsonii subsp*.berkhoffii, B. koehlerae*, *B. clarridgeiae*, *B. elizabethae*, *B. washoensis*, *B. quintana*, *B. bovis*, *B. volans*-like, and *B. rochalimae* [31,32,33,34,35]. Due to the symptomology of infected dogs, it is hypothesized that dogs are likely accidental hosts of *Bartonella* species. Infections with these pathogens result in endocarditis, myocarditis, vasculitis, and granulomatous disease in dogs, similar to the disease caused in humans [36,37].

Although a majority of these infections are thought to be accidental, dogs are most likely a reservoir of a few species of *Bartonella* including *B. henselae,* and *B. vinsonii subsp. Berkhoffii* [34]. *Bartonella* has been isolated from stray and domestic dogs in Chile, Sri Lanka, Brazil, and Columbia, with asymptomatic dogs exhibiting high infection rates with *B. henselae* and *B. vinsonii subsp. berkhoffii* [31,32,33]. 

The transmission of *Bartonella* between dogs is probably through vectors such as fleas and ticks [33,38,39]. DNA from *B. bovis*, *B. rochalimae*, *B chomelii*, *B. henselae*, *B. phoceensis*, *B. queensladensis*, *B. rattimassiliensis*, and *B. elizabethae* has been isolated from ticks removed from dogs [38,39]. Not much evidence exists to support a direct transmission from dogs to humans. However, a case in which a veterinarian and his daughter were infected with *B. vinsonii* subsp. *berkhoffii* along with their dog was documented [24]. The father and daughter experienced neurological symptoms and weight loss, and once antibiotics were administered, the symptoms ceased [24]. Although there is no direct evidence that dogs can transmit *Bartonella* to humans, *B. henselae*, *B. bovis*, *B. quintana*, and *B. vinsonii* subsp. *berkhoffii* DNA have been isolated through PCR in dog saliva [40]. Therefore, while *Bartonella* DNA has been detected in saliva, more evidence is needed to substantiate the notion of a direct transmission from dogs to humans. The most likely mode of transmission is through a vector such as fleas or ticks.

### 2.3. Other Domestic Animals

In 2007, a novel study found that two horses were infected with *B. henselae* [41]. Since then, *Bartonella* infection has been investigated through an experimental infection and a naturally occurring infection in horses [42,43]. When infected experimentally, *B. henselae*-infected horses developed acute bacteremia with no long-term effects and *B. bovis*-infected horses mostly were unaffected (1 horse had acute bacteremia) [42]. Subsequent to these studies, healthy and sick horses have been investigated for the presence of other *Bartonella* spp. Of note, *B. henselae*, *B. vinsonii* subsp. *berkhoffii*, and novel *Bartonella* have been identified in horses by utilizing an enrichment culture developed by the Breitschwerdt lab [43]. Transmission may be occurring through biting flies, ticks, and lice [43]. Since the listed *Bartonella* spp. found in horses cause disease in humans, horses may be accidental hosts and may potentially aid in the spread of infection through arthropod vectors.

Cattle are reservoirs of *B. bovis*, *B. schoenbuchensis*, and *B. chomelii* [44,45,46,47]. None of the aforementioned *Bartonella* spp. have been identified as human pathogens. The transmission between cattle occurs through many different vectors. In one study, *B. henselae* was identified in 12% of cows from Israel [44]. Although there is no evidence that a direct transmission is occurring, the potential exists for these reservoirs to transmit other yet unidentified *Bartonella* spp. 

Sheep and sheep keds (the sheep fly, *Melophagus ovinus*) are a reservoir and vector, respectively, of *B. melophagi* [48]. *B. melophagi* can only be found in domestic sheep species and does cause disease in humans [48,49]. It has been isolated from two patients, each having nonspecific abnormalities, including difficulty sleeping, muscle weakness, joint pain, and facial tremors [49]. An acute infection has been followed by reoccurring symptoms for up to 2 years after infection [49]. Although the strain has not been identified in many people, those working closely with animals such as horses, cattle, and sheep should be aware of Bartonellosis and the associated symptoms.

*B. alsatica* was first isolated from asymptomatic rabbits in 1999 [50]. The investigators injected an isolate into laboratory rabbits and observed bacteremia for 2–3 months, indicating rabbits as a reservoir [50]. Initially, the bacteria were isolated as a survey for reservoirs of *Bartonella* spp. However, since the discovery, *B. alsatica* has been identified in human disease cases. Two patients had blood-culture-negative endocarditis, and one patient developed lymphadenitis as a result of a *B. alsatica* infection [51,52,53]. All patients had close contact to rabbits before their symptoms began, implicating rabbits in the transmission cycle of *Bartonella* species. *Spilosyllus cuniculi* fleas collected from rabbits in France harbor *B. alsatica* DNA, are a likely vector between rabbits, and may be a vector that transmits *B. alsatica* to humans [54]. 

While serological surveys of nonhuman primates, either in the wild or in captive colonies, are limited, several studies in Asia have shown that macaques are susceptible to infection with *B. quintana* [55,56,57]. Nonhuman primates are thought to be a reservoir for this human pathogen, which was responsible for nearly one million cases of trench fever among soldiers in World War I. Phylogenetic analyses on *Bartonella* found in infected macaques further indicated that primates may serve as a natural host and that *B. quintana* may primarily be a zoonotic infection [58]. In these studies of infected macaques, an overt disease was not evident, though the pathogen persisted in the blood, consistent with their probable role as natural hosts.

## 3. Vectors for *Bartonella* Species

Numerous different vectors transmit *Bartonella* species. These include fleas, keds, lice, and sand flies and potentially ticks, mites, and spiders [4]. In the following section, the evidence of transmission as well as the prevalence of these vectors in the wild will be discussed.

### 3.1. Fleas and Lice

In 1996, it was determined that the cat flea, *Ctenocephalides felis*, was responsible for the transmission of *Bartonella henselae* between cats [26]. Fleas were removed from bacteremic cats and placed on specific pathogen-free kittens, which all became bacteremic 2 weeks after flea placement, except one that became bacteremic 6 weeks after placement [26]. To demonstrate that transmission did not occur directly between cats, infected cats were caged with specific pathogen-free kittens for 21 days, and the specific pathogen-free kittens did not develop bacteremia despite playing (biting and scratching) occurring between the cats [26]. *C. felis* has been implicated in the transmission of other *Bartonella* species such as *B. quintana*, *B. clarridgeiae*, and *B. koehlerae* [59,60,61]. *B. quintana* was also culturable from flea feces, implicating this vector in the transmission of trench fever, although the fleas were fed on infected blood, not representing a natural infection [59]. 

Other flea species have also been associated with *Bartonella* transmission, in particular, rat fleas. *Xenopsylla sp.* fleas collected from Palestine, Rwanda, Thailand, and in the US (California) can be infected with *Bartonella* species [61,62,63,64]. The *Leptopsylla taschenbergi*, fleas associated with small mammals, have been collected from the wild and are infected with *Bartonella* species as well [65]. *Ctenophthalmus nobilis*, rodent fleas collected from an area with a high *Bartonella* prevalence, experimentally transmitted *B. taylorii* and *B. grahamii* to *Bartonella*-free bank voles [66]. Links between rats and *Bartonella* are important to observe; as humans colonize more discrete areas of the world, the emergence of vector-borne disease increases due to our arrival into the life cycle of these pathogens.

Lice have been documented as transmitting *Bartonella quintana* since the 1920s, after World War I [67]. Many soldiers contracted trench fever in World War I and in World War II as well. The disease is found in unsanitary environments, where bathing infrequently allows body lice to serve as vectors. A reemergence of trench fever has been documented in homeless populations around the world in countries such as Colombia, Algeria, and France and in cities of the United States such as Washington D.C., San Francisco, and Seattle [68,69,70,71,72,73]. The disease is mostly associated with body lice. Green fluorescent protein-expressing *B. quintana* bacteria as an experimental system were found to replicate in the louse gut, and viable bacteria were also found in the feces of *Pediculus humanus* after the lice fed on an infected rabbit [74]. The role of head lice in transmitting *B. quintana* is less well-understood. In Africa, head and body lice were collected from 37 mono-infested individuals. The findings showed that 48 of 143 body lice and only 6 of 31 head lice harbored *B. quintana* [75]. One report showed that head lice could be experimentally infected and that *B. quintana* was found in the feces, but there was a higher amount of viable *B. quintana* in body lice than in head lice [76]. Most humans become infected with *B. quintana* due to louse feces, so the smaller frequency of infection caused by head lice makes sense, with fewer viable bacteria present in head lice feces. While fleas and lice are well-established vectors for transmitting different strains of *Bartonella*, increasing evidence links many other vectors to *Bartonella* spp. 

### 3.2. Arachnids (Spiders and Ticks)

Over the last 10 years, the topic of ticks transmitting *Bartonella* species has been widely debated. Evidence exists to support the transmission of *Bartonella* through many different species of ticks. *Ixodid* ticks, also known as hard ticks, appear to be the main type of tick associated with these bacteria. Tick cell lines have been used to show that *Bartonella* can replicate and survive within *Amblyoma americanum*, *Rhipicephalus sanguineus*, and *Ixodes scapularis* cells [77]. In California, questing ticks of *Ixodes pacificus*, *Dermacentor occidentalis*, and *Dermacentor variabilis* were collected when in the adult and nymphal stages and tested for *Bartonella* by PCR for the citrate synthase gene. [78]. All types of ticks were found to contain *Bartonella* DNA, although in varying percentages and locations. These data alone do not prove that ticks can transmit *Bartonella* spp. Bacteria; however, the results do show *Bartonella* DNA occurring naturally in these wild ticks. In Palestine, *Hyalomma* spp., *Haemphysalis* spp., and *Rhipicephalus* spp. ticks were collected from domestic animals and tested by PCR for the *Bartonella* intergenic transcribed spacer (ITS) region [38]. These ticks were infected with 4 strains of *Bartonella*: *B. rochalimae*, *B. chomelii*, *B. bovis*, and *B. koehlerae* [38]. While this study tested a collection of ticks found on domestic animals, the results suggest that individuals in close contact with these animals should be aware of the potential for transmission through tick bites. 

In a sampling of ticks (*Ixodes scapularis* and *Dermacentor variabilis*) and rodents (*Peromyscus leucopus*) from southern Indiana, the midgut contents of the tick species and rodent blood were analyzed by 16S sequencing. *Bartonella* was present in a moderate percentage (26% in *D. variabilis* and 13.3% in *I. scapularis*) of larvae and nymphs of both tick species, even those scored as unengorged, but was present in the majority (97.8%) of the rodents tested [79]. A survey of ticks from 16 states in the U.S. revealed that the overall prevalence of *Bartonella henselae* in *Ixodes* ticks was 2.5% [80]. Interestingly, the highest rate of both *Borrelia* spp. (63.2%) and *B. henselae* (10.3%) was found in *Ixodes affinis* ticks collected from North Carolina. *Ixodes ricinus* has been the focus of studies that support tick transmission of *Bartonella* spp. in Europe. This is because *I. ricinus* is an important vector for tick-borne diseases in Europe [81]. *I. ricinus* have been collected in the larval, nymphal, and adult stages in Austria [82]. The analyses revealed that 2.1% of all ticks were infected with *Bartonella* spp., with the highest rate in ticks derived from Vienna (with a 7.5% infection rate), and that adult ticks had a higher prevalence than other stages [82]. *B. henselae*, *B. doshiae*, and *B. grahamii* DNA were amplified, and this was the first study to find *Bartonella*-infected ticks in Austria [82]. A recent One Health perspective review on *Bartonella* indicated that the overall presence of *Bartonella* in ticks (combining evidence from multiple surveillance studies) was approx. 15% [83]. *B. henselae* DNA has also been isolated from *I. ricinus* removed from an infected cat. However, whether the cat gave the tick *Bartonella* or vice versa cannot be established, so the vector competence of these ticks for transmission cannot be determined [30]. A lab in France has studied the relationship between *I. ricinus* and *Bartonella* transmission. One study focused on the ability of ticks to maintain infection from one life stage to the next and tested a vertical transmission from adults to eggs. The authors used *B. henselae* and found that a transstadial transmission was possible and that a transovarial transmission was not likely [84]. The researchers also supplied evidence to support the vector competency of *I. ricinus* by amplifying *B. henselae* DNA from the salivary glands of infected ticks and by amplifying DNA from blood 72 h after infected ticks fed through an artificial system [84]. Although the evidence strongly suggests the ability of ticks to transmit these bacteria, the system employed artificial means for feeding; therefore, one major critique has been that it is not representative of a natural blood meal from a host. To address this issue, another experiment was performed to the assess vector competency of *I. ricinus* to transmit *Bartonella birtlesii* [85]. Mice were infected with *B. birtlesii* through an intravenous injection via a tail vein, and once mice were infected, naïve ticks were fed on the mice and kept for 3 months to molt. Nymphal ticks were shown to transmit *B. birtlesii* to naïve mice, and adult ticks were shown to infect blood through a feeder method [85]. *B. birtlesii* was identified in the blood of the recipient mice through PCR and immunofluorescence [85]. This evidence strongly supports the transmission of these bacteria by ticks. However, the limitation is that this only supports *I. ricinus’* ability to transmit a very specific strain of *Bartonella*, *B. birtlesii*, which is not linked to human disease. Concerns such as these related to vector competence and transmission can only be quelled by repeated studies utilizing multiple strains of *Bartonella* and differing tick species. 

An interesting case study provided evidence of spiders transmitting *Bartonella*. A mother and two sons suffered from neurological symptoms following bites suspected from woodlouse hunter spiders [18]. *Bartonella henselae* DNA was amplified from the blood of the family as well as from a woodlouse and a woodlouse hunter spider near the family’s home [18]. It cannot be determined if the family contracted the bacteria from the woodlouse or the woodlouse hunter spider or if the lice and spiders contracted the bacteria from the family. This case study points to the importance for diagnosticians to test for bacterial infections after suspected arachnid bites. It also emphasizes the lack of knowledge on the possible vectors that transmit *Bartonella* as well as the range of manifestations by infection with *Bartonella*. 

## 4. *Bartonella* in the Wild (Reservoirs)

### 4.1. Rodents

Rats have been closely associated with human zoonotic diseases. Urban communities provide excellent niches for rat survival, with access to many resources through contact with humans [86]. Rats have links to zoonotic infections such as plague and have vectors associated with this disease, mainly *Xenopsylla cheopsis*, the oriental rat flea [87]. However, rats can be infested with many ectoparasites such as lice, fleas, mites, and ticks, implicating them in transmission cycles of many diseases. These links have led researchers to investigate rodents and their common ectoparasites in the transmission of *Bartonella* spp.

*Bartonella elizabethae* complex *sensu lato* is closely associated with different rat species [88]. A phylogeographic analysis utilizing and comparing the citrate synthase gene of *Bartonella* to rats and other rodents found that *Bartonella* originated in Southeast Asia and a dispersal of the bacteria was due to rats and other rodents [88]. *Bartonella elizabethae* has been identified as an agent in culture-negative endocarditis in humans since 1993 [89]. In 1996, 33% of surveyed intravenous drug users in Baltimore, MD had antibodies to *B. elizabethae* [90]. This indicates a risk among certain populations in the United States and potentially other regions of the world.

In Thailand, several studies have been performed on the persistence of a *Bartonella* infection in rats, in their ectoparasites, and in humans by testing serum samples. In 2004, a survey of wild rodents showed an 8.7% infection rate and the researchers identified a novel *Bartonella* species with a close relation to *B. elizabethae* [91]. In 2015, another group identified *Bartonella* DNA in 17% of rodents, with a high prevalence in *Bandicota* spp. and *Rattus* spp. rats [63]. In the latter study, ectoparasites from the rats were also collected and the results showed that 57.1% of lice and 25.8% of collected fleas possessed *Bartonella* DNA; a lower prevalence was found in ticks (3.5%), and mites (1.7%) [63]. It was also noted that rats in areas of high ectoparasite numbers had a higher prevalence of *Bartonella* infection rates. This is important to distinguish because the route of transmission from rats to humans is poorly understood in *Bartonella* infections but likely is due to a vector. 

A serological survey in Thailand found 20 out of 261 human samples contained *Bartonella* DNA [92]. After an amplification of the citrate synthase gene, two patients were identified as actively infected with *B. henselae* [92]. The remaining patients were infected with *Bartonella* containing unique sequences, with a close relation to *B. elizabethae*, *B. tribocorum*, *B. rattimassiliensis*, *B. vinsonii subsp. arupensis*, and *B. tamiae* [92]. *Bartonella tamiae* was first sequenced from humans exhibiting febrile illness and has since been sequenced from chiggers removed from rats and directly from rats [93,94]. 

Other areas of the world have surveyed rodent populations for the presence of *Bartonella* spp. bacteria. In the country of Georgia, a woman developed lymphadenopathy and fever due to a *Bartonella* species related to *B. tribocorum* and *B. elizabethae* [95]. When the local rodent population was surveyed, 41.2% were found to harbor *Bartonella* DNA and 37.2% were positive by culture [96]. In Kenya, a staggering 60% of *Rattus* spp. rats in urban populations had *Bartonella*, whereas only 13% rats in a rural location had *Bartonella* [97]. All the data implicate rodents in the transmission cycle of *Bartonella* to humans, with rats in particular. However, much knowledge regarding the means of transmission from rodents to humans remains unknown. The relationship of rodents and the transmission of *Bartonella* should be studied closer and more thoroughly with controlled experiments to determine the exact routes of transmission between rodents, the transmission between rodents and their vectors, as well as the transmission between rodents to humans to determine risk of *Bartonella* infections to humans. 

### 4.2. Bats

Bats are common mammals that have a large geographic distribution, with links to emerging pathogens [98]. Specifically, their link to viral pathogens is understood, but their link to emerging bacterial pathogens is less well-characterized [99]. *Bartonella mayotimonensis*, which caused culture-negative endocarditis in a male patient, has been linked to bats [100,101]. Since this occurrence, more studies have been conducted to investigate the relationship between bats and *Bartonella* spp. 

*Bartonella* isolated from bats in Georgia were sequenced, and analyses of homology identified strains related to those isolated from dogs in Thailand and a relationship to strains isolated from humans in Poland [99]. Ectoparasites, such as ticks and bat flies, are thought to serve as a primary route of transmission between bats [99]. *Bartonella* has also been isolated from bats in France, Spain, Brazil, Argentina, Thailand, Romania, Hungary, and Nigeria [98,102,103,104,105,106]. 

Bat flies, in particular, are implicated as the most likely vector transmitting *Bartonella* between bats. However, studies have shown that, although bat flies do test positive for *Bartonella* DNA, the strains typically differ from those isolated from bat host populations [107]. The genetic diversity of bat flies and the *Bartonella* sp. isolated from these ectoparasites supports a shared evolution and suggests that a horizontal transmission may be occurring [106]. 

One interesting study determined a link between bats and humans. Twice a year, an African population goes into a bat cave to collect bats for consumption [98]. Bats and bat flies were collected and sequenced for *Bartonella* prevalence, and human populations in the surrounding area were also surveyed for *Bartonella* infections. A novel species, named *Bartonella rousetti*, was isolated from bats and bat flies. In addition, 8 out of 204 persons surveyed were seroreactive to *B. rousetti* [98]. This study implicates bats as a potential reservoir for human *Bartonella* infections, although the direct link to human disease is unclear. 

## 5. *Bartonella* as a Coinfection in Humans

Equivalent to the debate on whether ticks transmit *Bartonella*, there has been more evidence gathered to support coinfection of *Bartonella* with other vector-borne pathogens. Most evidence has occurred through serological surveys conducted in an effort to estimate the incidence of human coinfection. Several studies have also focused on the detection of pathogens within questing ticks. Although it is difficult to determine whether these coinfections occurred with one vector or transmission event or over the course of multiple events, one aspect is clear: A coinfection with these pathogens leads to difficulty clearing either infection and the antibiotic treatment should differ for individuals infected with multiple pathogens. 

In a Peruvian retrospective study, 35% of patients with Carrion’s disease had coinfections with *B. bacilliformis*, including *Salmonella* spp., *Shigella dysenteriae*, *Staphlococcus aureus*, *Enterobacter* spp., *Toxoplasma* spp., *Histoplasma* spp., *Pneumocystis carinii*, and *Plasmodium vivax* malaria [108]. Although most patients responded to antibiotic treatments, 4 patients with coinfections died [108]. Another study in Peru found that 37.5% of Bartonellosis patients in an outbreak were infected with *B. bacilliformis* and *Mycobacterium* spp. [109]. While not necessarily co-transmitted, these infectious complications can exacerbate disease. These data help to demonstrate the importance of monitoring coinfections with pathogens such as *Bartonella*. 

One serological survey conducted among the homeless in Columbia found 13.1% of enrolled individuals had IgG to both *Bartonella* spp. and *Rickettsia typhi* [68]. Lice collected from these homeless individuals were also screened, and 28% of louse pools were positive for *Bartonella* spp., while none were positive for *Rickettsia* spp., implicating other factors in this particular coinfection among the homeless in Bogotá [68]. From a collection of questing ticks surveyed in Germany, 15/104 nymphs were found to be coinfected with *Bartonella* spp. and *Rickettsia* spp. [110]. A study of volunteer blood donors in Namibia identified a high rate of exposure to *Coxiella burnetii*, Spotted Fever Group and Typhus Group *Rickettsiae*, and *Bartonella henselae*. While *B. henselae* exposure (2.9%) was the rarest of the those tested, over 20% of donors had been exposed to two or more pathogens, which was positively correlated with occupations involving animals [111]. Although there is no direct evidence, these pathogens were transmitted at one time, and flea and tick vectors transmit the pathogens; *Coxiella burnetii*, which causes Q fever in humans, in particular, could produce more serious complications as a coinfection [112]. In 2012, a survey of 39 farmers, 119 foresters, and 32 healthy blood donors (controls) was conducted to investigate an exposure to tick-borne pathogens and coinfections including *Borrelia burgdorferi*, *Anaplasma phagocytophilum*, *Bartonella* spp., and *Babesia microti* [113]. The sera obtained indicated that 23.1% of foresters, 27.7% of farmers, and 37.5% of control groups had antibodies to *Bartonella* [113]. A coinfection risk was directly linked to occupational exposure, and the highest rates of coinfection were with *Bartonella* and *B. burgdorferi* with 9.2% of forestry workers and 7.7% of farmers coinfected. The other coinfections observed were *Bartonella* spp. and *A. phagocytophilum* (1.7% forestry workers) and *Bartonella* spp. and *B. microti* (0.8% forestry workers). A small percentage (1.3%) of forestry workers even experienced triple infections with *Borrelia burgdorferi, Bartonella* spp., and *A. phagocytophilum*, and one individual had a quadruple infection with all pathogens investigated [113]. These data support *Bartonella* coinfections in humans with multiple pathogens transmitted by a variety of vectors.

In 2006, a study conducted in New Jersey found that out of 168 questing *Ixodes scapularis* ticks collected, 6.55% were infected with *Bartonella henselae* and 1.19% were coinfected with *B. henselae* and *Borrelia burgdorferi* [114]. Interestingly, there were 3 reported cases of individuals coinfected with *B. henselae* and *B. burgdorferi* in New Jersey in 2001 [115]. *I. scapularis* ticks were obtained from one of the patient’s household and tested positive for *B. henselae* and *Borrelia burgdorferi* DNA using PCR [115]. These patients all had neuroborreliosis, and after a treatment with antibiotics, their symptoms did not improve. However, once diagnosed as coinfected and placed on a more potent antibiotic regimen, the symptoms improved [115]. There is no direct evidence that the patients described acquired the infections simultaneously. Nevertheless, patients treated for Lyme disease should be examined for existing coinfections prior to antibiotic therapy. An initial discovery of coinfection could lead to an improved patient outcome.

In Europe, *I. ricinus* ticks transmit many diseases. Studies have been conducted whereby questing ticks were collected and serological data was analyzed in regions to determine the risk for coinfections with *Borrelia*. In France in 2011, a survey showed that, with a 32% *I. ricinus* rate of infection with *B. burgdorferi*, only about 0.1% demonstrated a coinfection with *Bartonella*, which was identified to be *B. birtlesii* [116]. In other parts of Europe, such as Germany, as much as 6.9% of the *I. ricinus* ticks were found to be infected with *Bartonella* and 25% of those ticks were coinfected with *Borrelia burgdorferi* [117]. A survey in Poland, on the other hand, found roughly 1.6% of *I. ricinus* ticks collected to be coinfected with *Bartonella henselae* and *Borrelia burgdorferi* [118]. Most recently, a serological analysis of more than 400 Lyme patient samples revealed that most patients possess antibodies to multiple tick-transmitted pathogens [119]. Depending on the Lyme disease patient category, between 15–33% were also seropositive for *Bartonella henselae*. These data support the possibility of a coinfection through a vector such as ticks. However, the occurrence of infections could have been either simultaneous or consecutive. 

In summary, the prevalence of *Bartonella* appears to be very broad, as these pathogens can utilize multiple vectors and cam infect a diverse range of hosts. Given the complex clinical manifestations and difficulty in effective treatments, the impact of these bacteria on human health may be more significant than is currently appreciated. These factors warrant further research on *Bartonella* prevalence, risks for infection, and pathobiology in mammalian hosts. 

## Figures and Tables

**Table 1:** The known *Bartonella* species, their hosts, and their vectors: The table has been adapted from Breitschwerdt, 2017 ^5^.

Bartonella Species	Host (s)	Vector(s)
*B. henselae*	Cat, human, dogs, horses	Fleas, lice, ticks, spiders
*B. quintana*	Humans, macaques, cats, dogs	Human body lice, fleas, bed bugs
*B. bacilliformis*	Humans	Sandflies, fleas
*B. koehlerae*	Cats, dogs, humans	Fleas
*B. vinsonii* ssp. *berkhoffi*	Dogs, horses, foxes, humans	Fleas, ticks
*B. bovis*	Cattle, cats, dogs, human	Biting flies, ticks
*B. clarridgeiae*	Cats, dogs	Fleas, ticks
*B. rattimassiliensis*	Rats	Fleas
*B. tamiae*	Rats, humans	Mites
*B. tribocorum*	Rats	Fleas
*B. rousetii*	Bats	Bat flies
*B. schoenbuchensis*	Cattle	Biting flies, ticks
*B. chomelii*	Cattle	Biting flies, ticks
*B. doshiae*	Rats, humans	Fleas
*B. grahamii*	Mice, humans	Fleas
*B. birtlesii*	Mice	Fleas
*B. mayotimonensis*	Bats, humans	Bat flies, fleas, ticks
*B. elizabethae*	Rats, humans, dogs	Fleas
*B. washoensis*	Dogs, humans	Fleas, ticks
*B. rochalimae*	Dogs, humans	Fleas, ticks
*B. vinsonii* ssp. *arupensis*	Dogs, humans	Fleas, ticks
*B.melophagi*	Sheep, humans	Sheep keds
*B. alsatica*	Rabbits, humans	Fleas, ticks

## References

[1] Angelakis E., Raoult D. (2014). Pathogenicity and treatment of *bartonella* infections. Int. J. Antimicrob. Agents.

[2] Chomel B.B., Kasten R.W., Williams C., Wey A.C., Henn J.B., Maggi R., Carrasco S., Mazet J., Boulouis H.J., Maillard R. (2009). *Bartonella* endocarditis: A pathology shared by animal reservoirsand patients. Ann. N. Y. Acad. Sci..

[3] Iannino F., Salucci S., Di Provvido A., Paolini A., Ruggieri E. (2018). *Bartonella* infections in humans dogs and cats. Vet. Ital..

[4] Breitschwerdt E.B. (2017). Bartonellosis, one health and all creatures great and small. Vet. Dermatol..

[5] Ben-Tekaya H., Gorvel J.P., Dehio C. (2013). *Bartonella* and Brucella—Weapons and strategies for stealth attack. Cold Spring Harb. Perspect. Med..

[6] Breitschwerdt E.B., Greenberg R., Maggi R.G., Mozayeni B.R., Lewis A., Bradley J.M. (2019). *Bartonella henselae* bloodstream infection in a boy with pediatric acute-onset neuropsychiatric syndrome. J. Cent. Nervous Syst. Dis..

[7] Kalogeropoulos D., Asproudis I., Stefaniotou M., Moschos M.M., Mentis A., Malamos K., Kalogeropoulos C. (2019). *Bartonella* henselae- and quintana-associated uveitis: A case series and approach of a potentially severe disease with a broad spectrum of ocular manifestations. Int. Ophthalmol..

[8] Breitschwerdt E.B., Mascarelli P.E., Schweickert L.A., Maggi R.G., Hegarty B.C., Bradley J.M., Woods C.W. (2011). Hallucinations, sensory neuropathy, and peripheral visual deficits in a young woman infected with *bartonella* koehlerae. J. Clin. Microbiol..

[9] Gurfield A.N., Boulouis H.J., Chomel B.B., Heller R., Kasten R.W., Yamamoto K., Piemont Y. (1997). Coinfection with *bartonella* clarridgeiae and *bartonella* henselae and with different *bartonella* henselae strains in domestic cats. J. Clin. Microbiol..

[10] Balakrishnan N., Ericson M., Maggi R., Breitschwerdt E.B. (2016). Vasculitis, cerebral infarction and persistent *bartonella* henselae infection in a child. Parasites Vectors.

[11] Mabra D., Yeh S., Shantha J.G. (2018). Ocular manifestations of bartonellosis. Curr. Opin. Ophthalmol..

[12] Marques L.C., Pincerato K., Yoshimura A.A., Andrade F.E.M., Barros A. (2018). Cat scratch disease presenting as axillary lymphadenopathy and a palpable benign mammary nodule mimicking a carcinoma. Revista da Sociedade Brasileira de Medicina Tropical.

[13] Markaki S., Sotiropoulou M., Papaspirou P., Lazaris D. (2003). Cat-scratch disease presenting as a solitary tumour in the breast: Report of three cases. Eur. J. Obstet. Gynecol. Reproduct. Biol..

[14] Povoski S.P., Spigos D.G., Marsh W.L. (2003). An unusual case of cat-scratch disease from *bartonella* quintana mimicking inflammatory breast cancer in a 50-year-old woman. Breast J..

[15] Mosbacher M.E., Klotz S., Klotz J., Pinnas J.L. (2011). *Bartonella* henselae and the potential for arthropod vector-borne transmission. Vector-Borne Zoonotic Dis..

[16] Guru P.K., Agarwal A., Fritz A. (2018). A miraculous recovery: *Bartonella* henselae infection following a red ant bite. BMJ Case Rep..

[17] Schouls L.M., Van De Pol I., Rijpkema S.G.T., Schot C.S. (1999). Detection and identification of ehrlichia, borrelia burgdorferi sensu lato, and *bartonella* species in dutch ixodes ricinus ticks. J. Clin. Microbiol..

[18] Mascarelli P.E., Maggi R.G., Hopkins S., Mozayeni B.R., Trull C.L., Bradley J.M., Hegarty B.C., Breitschwerdt E.B. (2013). *Bartonella* henselae infection in a family experiencing neurological and neurocognitive abnormalities after woodlouse hunter spider bites. Parasites Vectors.

[19] Leulmi H., Bitam I., Berenger J.M., Lepidi H., Rolain J.M., Almeras L., Raoult D., Parola P. (2015). Competence of cimex lectularius bed bugs for the transmission of *bartonella* quintana, the agent of trench fever. PLoS Negl. Trop. Dis..

[20] Kordick D.L., Wilson K.H., Sexton D.J., Hadfield T.L., Berkhoff H.A., Breitschwerdt E.B. (1995). Prolonged *bartonella* bacteremia in cats associated with cat-scratch disease patients. J. Clin. Microbiol..

[21] Droz S., Chi B., Horn E., Steigerwalt A.G., Whitney A.M., Brenner D.J. (1999). *Bartonella* koehlerae sp. Nov., isolated from cats. J. Clin. Microbiol..

[22] Margileth A.M., Baehren D.F. (1998). Chest-wall abscess due to cat-scratch disease (csd) in an adult with antibodies to *bartonella* clarridgeiae: Case report and review of the thoracopulmonary manifestations of CSD. Clin. Infect. Dis..

[23] Avidor B., Graidy M., Efrat G., Leibowitz C., Shapira G., Schattner A., Zimhony O., Giladi M. (2004). *Bartonella* koehlerae, a new cat-associated agent of culture-negative human endocarditis. J. Clin. Microbiol..

[24] Breitschwerdt E.B., Maggi R.G., Lantos P.M., Woods C.W., Hegarty B.C., Bradley J.M. (2010). *Bartonella* vinsonii subsp. Berkhoffii and *bartonella* henselae bacteremia in a father and daughter with neurological disease. Parasites Vectors.

[25] Chomel B.B., Kasten R.W., Stuckey M.J., Breitschwerdt E.B., Maggi R.G., Henn J.B., Koehler J.E., Chang C.C. (2014). Experimental infection of cats with afipia felis and various *bartonella* species or subspecies. Vet. Microbiol..

[26] Chomel B.B., Kasten R.W., Floyd-Hawkins K., Chi B., Yamamoto K., Roberts-Wilson J., Gurfield A.N., Abbott R.C., Pedersen N.C., Koehler J.E. (1996). Experimental transmission of *bartonella* henselae by the cat flea. J. Clin. Microbiol..

[27] Abbott R.C., Chomel B.B., Kasten R.W., Floyd-Hawkins K.A., Kikuchi Y., Koehler J.E., Pedersen N.C. (1997). Experimental and natural infection with *bartonella* henselae in domestic cats. Comp. Immunol. Microbiol. Infect. Dis..

[28] Oskouizadeh K., Zahraei-Salehi T., Aledavood S. (2010). Detection of *bartonella* henselae in domestic cats’ saliva. Iran. J. Microbiol..

[29] Breitschwerdt E.B., Maggi R.G., Sigmon B., Nicholson W.L. (2007). Isolation of *bartonella* quintana from a woman and a cat following putative bite transmission. J. Clin. Microbiol..

[30] Regier Y., Ballhorn W., Kempf V.A.J. (2017). Molecular detection of *bartonella* henselae in 11 ixodes ricinus ticks extracted from a single cat. Parasites Vectors.

[31] Perez C., Maggi R.G., Diniz P.P., Breitschwerdt E.B. (2011). Molecular and serological diagnosis of *bartonella* infection in 61 dogs from the United States. J. Vet. Intern. Med..

[32] Müller A., Soto F., Sepúlveda M., Bittencourt P., Benevenute J.L., Ikeda P., Machado R.Z., André M.R. (2018). *Bartonella* vinsonii subsp. berkhoffii and B. henselae in dogs. Epidemiol. Infect..

[33] Brenner E.C., Chomel B.B., Singhasivanon O.U., Namekata D.Y., Kasten R.W., Kass P.H., Cortes-Vecino J.A., Gennari S.M., Rajapakse R.P., Huong L.T. (2013). *Bartonella* infection in urban and rural dogs from the tropics: Brazil, Colombia, Sri Lanka and Vietnam. Epidemiol. Infect..

[34] Alvarez-Fernandez A., Breitschwerdt E.B., Solano-Gallego L. (2018). *Bartonella* infections in cats and dogs including zoonotic aspects. Parasites Vectors.

[35] Diniz P.P., Morton B.A., Tngrian M., Kachani M., Barrón E.A., Gavidia C.M., Gilman R.H., Angulo N.P., Brenner E.C., Lerner R. (2013). Infection of domestic dogs in peru by zoonotic *bartonella* species: A cross-sectional prevalence study of 219 asymptomatic dogs. PLoS Negl. Trop. Dis..

[36] Breitschwerdt E.B., Blann K.R., Stebbins M.E., Muñana K.R., Davidson M.G., Jackson H.A., Willard M.D. (2004). Clinicopathological abnormalities and treatment response in 24 dogs seroreactive to *bartonella* vinsonii (berkhoffii) antigens. J. Am. Anim. Hosp. Assoc..

[37] Friedenberg S.G., Balakrishnan N., Guillaumin J., Cooper E.S., Lewis K., Russell D.S., Breitschwerdt E.B. (2015). Splenic vasculitis, thrombosis, and infarction in a febrile dog infected with *bartonella* henselae. J. Vet. Emerg. Crit. Care.

[38] Ereqat S., Nasereddin A., Vayssier-Taussat M., Abdelkader A., Al-Jawabreh A., Zaid T., Azmi K., Abdeen Z. (2016). Molecular evidence of *bartonella* species in ixodid ticks and domestic animals in palestine. Front. Microbiol..

[39] Tsai Y.-L., Lin C.-C., Chomel B.B., Chuang S.-T., Tsai K.-H., Wu W.-J., Huang C.-G., Yu J.-C., Sung M.-H., Kass P.H. (2011). *Bartonella* infection in shelter cats and dogs and their ectoparasites. Vector-Borne Zoonotic Dis..

[40] Duncan A.W., Maggi R.G., Breitschwerdt E.B. (2007). Bartonella DNA in dog saliva. Emerg. Infect. Dis..

[41] Jones S.L., Maggi R., Shuler J., Alward A., Breitschwerdt E.B. (2008). Detection of bartonella henselae in the blood of 2 adult horses. J. Vet. Intern. Med..

[42] Palmero J., Pusterla N., Cherry N.A., Kasten R.W., Mapes S., Boulouis H.J., Breitschwerdt E.B., Chomel B.B. (2012). Experimental infection of horses with *bartonella* henselae and *bartonella* bovis. J. Vet. Intern. Med..

[43] Cherry N.A., Jones S.L., Maggi R.G., Davis J.L., Breitschwerdt E.B. (2012). *Bartonella* spp. Infection in healthy and sick horses and foals from the southeastern United States. J. Vet. Intern. Med..

[44] Gutiérrez R., Cohen L., Morick D., Mumcuoglu K.Y., Harrus S., Gottlieb Y. (2014). Identification of different *bartonella* species in the cattle tail louse (haematopinus quadripertusus) and in cattle blood. Appl. Environ. Microbiol..

[45] Chang C.C., Chomel B.B., Kasten R.W., Heller R.M., Kocan K.M., Ueno H., Yamamoto K., Bleich V.C., Pierce B.M., Gonzales B.J. (2000). *Bartonella* spp. Isolated from wild and domestic ruminants in north America. Emerg. Infect. Dis..

[46] Rolain J.M., Rousset E., La Scola B., Duquesnel R., Raoult D. (2003). *Bartonella* schoenbuchensis isolated from the blood of a French cow. Ann. N. Y. Acad. Sci..

[47] Maillard R., Riegel P., Barrat F., Bouillin C., Thibault D., Gandoin C., Halos L., Demanche C., Alliot A., Guillot J. (2004). *Bartonella* chomelii sp. nov., isolated from French domestic cattle (bos taurus). Int. J. Syst. Evol. Microbiol..

[48] Kosoy M., Bai Y., Enscore R., Rizzo M.R., Bender S., Popov V., Albayrak L., Fofanov Y., Chomel B. (2016). *Bartonella* melophagi in blood of domestic sheep (ovis aries) and sheep keds (melophagus ovinus) from the southwestern US: Cultures, genetic characterization, and ecological connections. Vet. Microbiol..

[49] Maggi R.G., Kosoy M., Mintzer M., Breitschwerdt E.B. (2009). Isolation of candidatus *bartonella* melophagi from human blood. Emerg. Infect. Dis..

[50] Heller R., Kubina M., Mariet P., Riegel P., Delacour G., Dehio C., Lamarque F., Kasten R., Boulouis H.J., Monteil H. (1999). *Bartonella* alsatica sp. nov., a new *bartonella* species isolated from the blood of wild rabbits. Int. J. Syst. Bacteriol..

[51] Raoult D., Roblot F., Rolain J.M., Besnier J.M., Loulergue J., Bastides F., Choutet P. (2006). First isolation of *bartonella* alsatica from a valve of a patient with endocarditis. J. Clin. Microbiol..

[52] Jeanclaude D., Godmer P., Leveiller D., Pouedras P., Fournier P.E., Raoult D., Rolain J.M. (2009). *Bartonella* alsatica endocarditis in a French patient in close contact with rabbits. Clin. Microbiol. Infect..

[53] Angelakis E., Lepidi H., Canel A., Rispal P., Perraudeau F., Barre I., Rolain J.M., Raoult D. (2008). Human case of *bartonella* alsatica lymphadenitis. Emerg. Infect. Dis..

[54] Kernif T., Parola P., Ricci J.C., Raoult D., Rolain J.M. (2010). Molecular detection of *bartonella* alsatica in rabbit fleas, France. Emerg. Infect. Dis..

[55] Li H., Liu W., Zhang G.Z., Sun Z.Z., Bai J.Y., Jiang B.G., Zhang Y.Y., Zhao X.G., Yang H., Tian G. (2013). Transmission and maintenance cycle of *bartonella* quintana among rhesus macaques, China. Emerg. Infect. Dis..

[56] Sato S., Kabeya H., Yoshino A., Sekine W., Suzuki K., Tamate H.B., Yamazaki S., Chomel B.B., Maruyama S. (2015). Japanese macaques (macaca fuscata) as natural reservoir of *bartonella* quintana. Emerg. Infect. Dis..

[57] Huang R., Liu Q., Li G., Li D., Song X., Birtles R.J., Zhao F. (2011). *Bartonella* quintana infections in captive monkeys, China. Emerg. Infect. Dis..

[58] Li H., Bai J.-Y., Wang L.-Y., Zeng L., Shi Y.-S., Qiu Z.-L., Ye H.-H., Zhang X.-F., Lu Q.-B., Kosoy M. (2013). Genetic diversity of *bartonella* quintana in macaques suggests zoonotic origin of trench fever. Mol. Ecol..

[59] Kernif T., Leulmi H., Socolovschi C., Berenger J.M., Lepidi H., Bitam I., Rolain J.M., Raoult D., Parola P. (2014). Acquisition and excretion of *bartonella* quintana by the cat flea, ctenocephalides felis felis. Mol. Ecol..

[60] Šlapeta J., Lawrence A., Reichel M.P. (2018). Cat fleas (ctenocephalides felis) carrying rickettsia felis and *bartonella* species in Hong Kong. Parasitol. Int..

[61] Nasereddin A., Risheq A., Harrus S., Azmi K., Ereqat S., Baneth G., Salant H., Mumcuoglu K.Y., Abdeen Z. (2014). *Bartonella* species in fleas from palestinian territories: Prevalence and genetic diversity. J. Vector Ecol. J. Soc. Vector Ecol..

[62] Nziza J., Tumushime J.C., Cranfield M., Ntwari A.E., Modry D., Mudakikwa A., Gilardi K., Slapeta J. (2019). Fleas from domestic dogs and rodents in rwanda carry rickettsia asembonensis and *bartonella* tribocorum. Med. Vet. Entomol..

[63] Klangthong K., Promsthaporn S., Leepitakrat S., Schuster A.L., McCardle P.W., Kosoy M., Takhampunya R. (2015). The distribution and diversity of *bartonella* species in rodents and their ectoparasites across Thailand. PLoS ONE.

[64] Billeter S.A., Gundi V.A., Rood M.P., Kosoy M.Y. (2011). Molecular detection and identification of *bartonella* species in xenopsylla cheopis fleas (siphonaptera: Pulicidae) collected from rattus norvegicus rats in Los Angeles, California. Appl. Environ. Microbiol..

[65] Cevidanes A., Altet L., Chirife A.D., Proboste T., Millan J. (2017). Drivers of *bartonella* infection in micromammals and their fleas in a mediterranean peri-urban area. Vet. Microbiol..

[66] Bown K.J., Bennet M., Begon M. (2004). Flea-borne *bartonella* grahamii and *bartonella* taylorii in bank voles. Emerg. Infect. Dis..

[67] Anstead G.M. (2016). The centenary of the discovery of trench fever, an emerging infectious disease of World War 1. Lancet Infect. Dis..

[68] Faccini-Martinez A.A., Marquez A.C., Bravo-Estupinan D.M., Calixto O.J., Lopez-Castillo C.A., Botero-Garcia C.A., Hidalgo M., Cuervo C. (2017). *Bartonella* quintana and typhus group rickettsiae exposure among homeless persons, Bogota, Colombia. Emerg. Infect. Dis..

[69] Louni M., Mana N., Bitam I., Dahmani M., Parola P., Fenollar F., Raoult D., Mediannikov O. (2018). Body lice of homeless people reveal the presence of several emerging bacterial pathogens in northern Algeria. PLoS Negl. Trop. Dis..

[70] Drali R., Sangare A.K., Boutellis A., Angelakis E., Veracx A., Socolovschi C., Brouqui P., Raoult D. (2014). *Bartonella* quintana in body lice from scalp hair of homeless persons, France. Emerg. Infect. Dis..

[71] Bonilla D.L., Cole-Porse C., Kjemtrup A., Osikowicz L., Kosoy M. (2014). Risk factors for human lice and bartonellosis among the homeless, San Francisco, California, USA. Emerg. Infect. Dis..

[72] Ghidey F.Y., Igbinosa O., Mills K., Lai L., Woods C., Ruiz M.E., Fishbein D., Sampath R., Lowery R., Wortmann G. (2016). Case series of *bartonella* quintana blood culture-negative endocarditis in Washington, DC. JMM Case Rep..

[73] Jackson L.A., Spach D.H., Kippen D.A., Sugg N.K., Regnery R.L., Sayers M.H., Stamm W.E. (1996). Seroprevalence to *bartonella* quintana among patients at a community clinic in downtown Seattle. J. Infect. Dis..

[74] Fournier P.E., Minnick M.F., Lepidi H., Salvo E., Raoult D. (2001). Experimental model of human body louse infection using green fluorescent protein-expressing *bartonella* quintana. Infect. Immun..

[75] Drali R., Shako J.C., Davoust B., Diatta G., Raoult D. (2015). A new clade of african body and head lice infected by *bartonella* quintana and yersinia pestis-democratic Republic of the Congo. Am. J. Trop. Med. Hyg..

[76] Kim J.H., Previte D.J., Yoon K.S., Murenzi E., Koehler J.E., Pittendrigh B.R., Lee S.H., Clark J.M. (2017). Comparison of the proliferation and excretion of *bartonella* quintana between body and head lice following oral challenge. Insect Mol. Biol..

[77] Billeter S.A., Diniz P.P., Battisti J.M., Munderloh U.G., Breitschwerdt E.B., Levy M.G. (2009). Infection and replication of *bartonella* species within a tick cell line. Exp. Appl. Acarol..

[78] Chang C.C., Hayashidani H., Pusterla N., Kasten R.W., Madigan J.E., Chomel B.B. (2002). Investigation of *bartonella* infection in ixodid ticks from California. Comp. Immunol. Microbiol. Infect. Dis..

[79] Rynkiewicz E.C., Hemmerich C., Rusch D.B., Fuqua C., Clay K. (2015). Concordance of bacterial communities of two tick species and blood of their shared rodent host. Mol. Ecol..

[80] Maggi R.G., Toliver M., Richardson T., Mather T., Breitschwerdt E.B. (2019). Regional prevalences of borrelia burgdorferi, borrelia bissettiae, and *bartonella* henselae in ixodes affinis, ixodes pacificus and ixodes scapularis in the USA. Ticks Tick Borne Dis..

[81] Cayol C., Jaaskelainen A., Koskela E., Kyrolainen S., Mappes T., Siukkola A., Kallio E.R. (2018). Sympatric ixodes-tick species: Pattern of distribution and pathogen transmission within wild rodent populations. Sci. Rep..

[82] Muller A., Reiter M., Schotta A.M., Stockinger H., Stanek G. (2016). Detection of *bartonella* spp. In ixodes ricinus ticks and *bartonella* seroprevalence in human populations. Ticks Tick-Borne Dis..

[83] Regier Y., O’Rourke F., Kempf V.A.J. (2016). *Bartonella* spp.—A chance to establish one health concepts in veterinary and human medicine. Parasites Vectors.

[84] Cotte V., Bonnet S., Le Rhun D., Le Naour E., Chauvin A., Boulouis H.J., Lecuelle B., Lilin T., Vayssier-Taussat M. (2008). Transmission of *bartonella* henselae by ixodes ricinus. Emerg. Infect. Dis..

[85] Reis C., Cote M., Le Rhun D., Lecuelle B., Levin M.L., Vayssier-Taussat M., Bonnet S.I. (2011). Vector competence of the tick ixodes ricinus for transmission of *bartonella* birtlesii. PLoS Negl. Trop. Dis..

[86] Himsworth C.G., Parsons K.L., Jardine C., Patrick D.M. (2013). Rats, cities, people, and pathogens: A systematic review and narrative synthesis of literature regarding the ecology of rat-associated zoonoses in urban centers. Vector Borne Zoonotic Dis. (Larchmont N.Y.).

[87] Fernandez-Gonzalez A.M., Kosoy M.Y., Rubio A.V., Graham C.B., Montenieri J.A., Osikowicz L.M., Bai Y., Acosta-Gutierrez R., Avila-Flores R., Gage K.L. (2016). Molecular survey of *bartonella* species and yersinia pestis in rodent fleas (siphonaptera) from Chihuahua, Mexico. J. Med. Entomol..

[88] Hayman D.T., McDonald K.D., Kosoy M.Y. (2013). Evolutionary history of rat-borne *bartonella*: The importance of commensal rats in the dissemination of bacterial infections globally. Ecol. Evol..

[89] Daly J.S., Worthington M.G., Brenner D.J., Moss C.W., Hollis D.G., Weyant R.S., Steigerwalt A.G., Weaver R.E., Daneshvar M.I., O’Connor S.P. (1993). Rochalimaea elizabethae sp. nov. Isolated from a patient with endocarditis. J. Clin. Microbiol..

[90] Comer J.A., Flynn C., Regnery R.L., Vlahov D., Childs J.E. (1996). Antibodies to *bartonella* species in inner-city intravenous drug users in Baltimore, MD. Arch. Intern. Med..

[91] Castle K.T., Kosoy M., Lerdthusnee K., Phelan L., Bai Y., Gage K.L., Leepitakrat W., Monkanna T., Khlaimanee N., Chandranoi K. (2004). Prevalence and diversity of *bartonella* in rodents of northern Thailand: A comparison with *bartonella* in rodents from southern China. Am. J. Trop. Med. Hyg..

[92] Kosoy M., Bai Y., Sheff K., Morway C., Baggett H., Maloney S.A., Boonmar S., Bhengsri S., Dowell S.F., Sitdhirasdr A. (2010). Identification of *bartonella* infections in febrile human patients from Thailand and their potential animal reservoirs. Am. J. Trop. Med. Hyg..

[93] Kosoy M., Morway C., Sheff K.W., Bai Y., Colborn J., Chalcraft L., Dowell S.F., Peruski L.F., Maloney S.A., Baggett H. (2008). *Bartonella* tamiae sp. Nov., a newly recognized pathogen isolated from three human patients from Thailand. J. Clin. Microbiol..

[94] Kabeya H., Colborn J.M., Bai Y., Lerdthusnee K., Richardson J.H., Maruyama S., Kosoy M.Y. (2010). Detection of *bartonella* tamiae DNA in ectoparasites from rodents in thailand and their sequence similarity with bacterial cultures from Thai patients. Vector Borne Zoonotic Dis. (Larchmont N.Y.).

[95] Kandelaki G., Malania L., Bai Y., Chakvetadze N., Katsitadze G., Imnadze P., Nelson C., Harrus S., Kosoy M. (2016). Human lymphadenopathy caused by ratborne *bartonella*, tbilisi, Georgia. Emerg. Infect. Dis..

[96] Malania L., Bai Y., Osikowicz L.M., Tsertsvadze N., Katsitadze G., Imnadze P., Kosoy M. (2016). Prevalence and diversity of *bartonella* species in rodents from Georgia (caucasus). Am. J. Trop. Med. Hyg..

[97] Halliday J.E., Knobel D.L., Agwanda B., Bai Y., Breiman R.F., Cleaveland S., Njenga M.K., Kosoy M. (2015). Prevalence and diversity of small mammal-associated *bartonella* species in rural and urban Kenya. PLoS Negl. Trop. Dis..

[98] Bai Y., Osinubi M.O.V., Osikowicz L., McKee C., Vora N.M., Rizzo M.R., Recuenco S., Davis L., Niezgoda M., Ehimiyein A.M. (2018). Human exposure to novel *bartonella* species from contact with fruit bats. Emerg. Infect. Dis..

[99] Urushadze L., Bai Y., Osikowicz L., McKee C., Sidamonidze K., Putkaradze D., Imnadze P., Kandaurov A., Kuzmin I., Kosoy M. (2017). Prevalence, diversity, and host associations of *bartonella* strains in bats from Georgia (caucasus). PLoS Negl. Trop. Dis..

[100] Lin E.Y., Tsigrelis C., Baddour L.M., Lepidi H., Rolain J.M., Patel R., Raoult D. (2010). Candidatus *bartonella* mayotimonensis and endocarditis. Emerg. Infect. Dis..

[101] Veikkolainen V., Vesterinen E.J., Lilley T.M., Pulliainen A.T. (2014). Bats as reservoir hosts of human bacterial pathogen, *bartonella* mayotimonensis. Emerg. Infect. Dis..

[102] Stuckey M.J., Boulouis H.J., Cliquet F., Picard-Meyer E., Servat A., Arechiga-Ceballos N., Echevarria J.E., Chomel B.B. (2017). Potentially zoonotic *bartonella* in bats from France and Spain. Emerg. Infect. Dis..

[103] Ikeda P., Seki M.C., Carrasco A.O.T., Rudiak L.V., Miranda J.M.D., Goncalves S.M.M., Hoppe E.G.L., Albuquerque A.C.A., Teixeira M.M.G., Passos C.E. (2017). Evidence and molecular characterization of *bartonella* spp. And hemoplasmas in neotropical bats in Brazil. Epidemiol. Infect..

[104] Cicuttin G.L., De Salvo M.N., La Rosa I., Dohmen F.E.G. (2017). Neorickettsia risticii, rickettsia sp. And *bartonella* sp. In tadarida brasiliensis bats from Buenos Aires, Argentina. Comp. Immunol. Microbiol. Infect. Dis..

[105] McKee C.D., Kosoy M.Y., Bai Y., Osikowicz L.M., Franka R., Gilbert A.T., Boonmar S., Rupprecht C.E., Peruski L.F. (2017). Diversity and phylogenetic relationships among *bartonella* strains from Thai bats. PLoS ONE.

[106] Sandor A.D., Foldvari M., Krawczyk A.I., Sprong H., Corduneanu A., Barti L., Gorfol T., Estok P., Kovats D., Szekeres S. (2018). Eco-epidemiology of novel *bartonella* genotypes from parasitic flies of insectivorous bats. Microb. Ecol..

[107] Moskaluk A.E., Stuckey M.J., Jaffe D.A., Kasten R.W., Aguilar-Setien A., Olave-Leyva J.I., Galvez-Romero G., Obregon-Morales C., Salas-Rojas M., Garcia-Flores M.M. (2018). Molecular detection of *bartonella* species in blood-feeding bat flies from mexico. Vector Borne Zoonotic Dis. (Larchmont N.Y.).

[108] Maguina C., Garcia P.J., Gotuzzo E., Cordero L., Spach D.H. (2001). Bartonellosis (carrion’s disease) in the modern era. Clin. Infect. Dis..

[109] Silva-Caso W., Mazulis F., Weilg C., Aguilar-Luis M.A., Sandoval I., Correa-Nunez G., Li D., Song X., Liu Q., Del Valle-Mendoza J. (2017). Co-infection with *bartonella* bacilliformis and mycobacterium spp. In a coastal region of Peru. BMC Res. Notes.

[110] Galfsky D., Krol N., Pfeffer M., Obiegala A. (2019). Long-term trends of tick-borne pathogens in regard to small mammal and tick populations from Saxony, Germany. Parasit Vectors.

[111] Noden B.H., Tshavuka F.I., van der Colf B.E., Chipare I., Wilkinson R. (2014). Exposure and risk factors to coxiella burnetii, spotted fever group and typhus group rickettsiae, and *bartonella* henselae among volunteer blood donors in Namibia. PLoS ONE.

[112] Maurin M., Raoult D. (1999). Q fever. Clin. Microbiol. Rev..

[113] Chmielewska-Badora J., Moniuszko A., Zukiewicz-Sobczak W., Zwolinski J., Piatek J., Pancewicz S. (2012). Serological survey in persons occupationally exposed to tick-borne pathogens in cases of co-infections with borrelia burgdorferi, anaplasma phagocytophilum, *bartonella* spp. And babesia microti. Ann. Agric. Environ. Med. AAEM.

[114] Holden K., Boothby J.T., Kasten R.W., Chomel B.B. (2006). Co-detection of *bartonella* henselae, borrelia burgdorferi, and anaplasma phagocytophilum in ixodes pacificus ticks from California, USA. Vector Borne Zoonotic Dis. (Larchmont N.Y.).

[115] Eskow E., Rao R.V., Mordechai E. (2001). Concurrent infection of the central nervous system by borrelia burgdorferi and *bartonella* henselae: Evidence for a novel tick-borne disease complex. Arch. Neurol..

[116] Reis C., Cote M., Paul R.E., Bonnet S. (2011). Questing ticks in suburban forest are infected by at least six tick-borne pathogens. Vector Borne Zoonotic Dis. (Larchmont N.Y.).

[117] Mietze A., Strube C., Beyerbach M., Schnieder T., Goethe R. (2011). Occurrence of *bartonella* henselae and borrelia burgdorferi sensu lato co-infections in ticks collected from humans in Germany. Clin. Microbiol. Infect..

[118] Sytykiewicz H., Karbowiak G., Werszko J., Czerniewicz P., Sprawka I., Mitrus J. (2012). Molecular screening for *bartonella* henselae and borrelia burgdorferi sensu lato co-existence within ixodes ricinus populations in central and eastern parts of Poland. Ann. Agric. Environ. Med. AAEM.

[119] Garg K., Merilainen L., Franz O., Pirttinen H., Quevedo-Diaz M., Croucher S., Gilbert L. (2018). Evaluating polymicrobial immune responses in patients suffering from tick-borne diseases. Sci. Rep..

